# EZH2 Regulates Lipopolysaccharide-Induced Periodontal Ligament Stem Cell Proliferation and Osteogenesis through TLR4/MyD88/NF-*κ*B Pathway

**DOI:** 10.1155/2021/7625134

**Published:** 2021-12-01

**Authors:** Pengcheng Wang, Huan Tian, Zheng Zhang, Zuomin Wang

**Affiliations:** ^1^Department of Stomatology, Beijing Shijitan Hospital, Capital Medical University, Beijing 100038, China; ^2^Department of Periodontology, Changsha Stomatological Hospital, Hunan University of Traditional Chinese Medicine, Changsha 410004, China; ^3^Department of Periodontology, Tianjin Stomatological Hospital and Tianjin Key Laboratory of Oral Function Reconstruction, Hospital of Stomatology, Nankai University, Tianjin 300041, China; ^4^Department of Stomatology, Beijing Chaoyang Hospital, Capital Medical University, Beijing 100020, China

## Abstract

**Background:**

Periodontitis induced by bacteria especially Gram-negative bacteria is the most prevalent chronic inflammatory disease worldwide. Emerging evidence supported that EZH2 plays a significant role in the inflammatory response of periodontal tissues. However, little information is available regarding the underlying mechanism of EZH2 in periodontitis. This study is aimed at determining the potential role and underlying mechanism of EZH2 in periodontitis.

**Methods:**

The protein levels of EZH2, H3K27ME, p-p65, p-IKB, TLR4, MyD88, Runx2, and OCN were examined by western blot assay. Proliferation was evaluated by CCK8 assay. The levels of TNF*α*, IL1*β*, and IL6 were detected by ELISA assay. Migration was detected by wound healing assay. The distribution of p65 was detected by immunofluorescence. The formation of mineralized nodules was analyzed using alizarin red staining.

**Results:**

LPS stimulation significantly promoted EZH2 and H3K27me3 expression in primary human periodontal ligament stem cells (PDLSCs). Targeting EZH2 prevented LPS-induced upregulation of the inflammatory cytokines and inhibition of cell proliferation and migration. Furthermore, EZH2 knockdown attenuated the TLR4/MyD88/NF-*κ*B signaling to facilitate PDLSC osteogenesis.

**Conclusions:**

Modulation of the NF-*κ*B pathway through the inhibition of EZH2 may offer a new perspective on the treatment of chronic apical periodontitis.

## 1. Introduction

Periodontitis is the common human chronic inflammatory disease of the tooth-surrounding tissues, which is associated with a series of systemic diseases [1, 2]. Periodontitis is accompanied by dysregulation or dysfunction of inflammation [3]. Exploring the induced inflammatory mechanism of chronic periodontitis may provide a theoretical foundation for periodontitis treatment.

Presently, controlling inflammation is the primary treatment for periodontitis. With the development of tissue engineering, periodontal regeneration therapy provides more possibilities. Tissue engineering consists of three elements, including “seed cells,” scaffold materials, and an external environment that facilitates cell growth and differentiation [4–6].

“Seed cells” is a key factor in the regeneration of periodontal tissue, which requires not only stable and mature differentiation function but also availability in clinical practice [5]. Periodontal ligament stem cells are isolated from periodontal root tissues after tooth extraction and can form “seed cells” in appropriate environment. Periodontal ligament stem cells have many characteristics of mesenchymal stem cells, such as self-renewal and multidirectional differentiation potential [7]. There have been in vitro studies that used periodontal ligament stem cells loaded on the constructed cell membrane to repair and regenerate damaged periodontal tissues [8]. There are also in vivo studies on the combination of autologous periodontal ligament stem cells and bovine bone mineral materials for the treatment of periodontal bone defects, which has good safety and clinical application prospects [9].

However, studies have shown that inflammation can affect the osteogenic ability of periodontal ligament stem cells [10], so it is particularly important to change the osteogenic ability of periodontal ligament stem cells under inflammatory microenvironment. Lipopolysaccharide (LPS) that constitutes the outer leaflet of the outer membrane of most Gram-negative bacteria participates in the immune responses of periodontitis [11, 12]. Li et al. reported that treatment with LPS and CF critically enhanced osteoclastogenesis in PDLSCs through the suppression of EphB4 and the induction of ephrinA2 signaling [13]. AlQranei et al. reported that TNF*α* secreted via LPS/TLR4 signaling regulated osteoclastogenesis in macrophages primed with RANKL and then treated with LPS [14].

This study is aimed at determining the potential role and underlying mechanism of EZH2 in periodontitis. Here, PDLSCs were used to induce periodontitis model through the LPS stimulation. We found that LPS stimulation significantly promoted EZH2 and H3K27me3 expression in PDLSCs. Targeting EZH2 prevented LPS-induced upregulation of TNF*α*, IL1*β*, and IL6 and proliferation and migration inhibition in PDLSCs. Furthermore, EZH2 knockdown inhibits TLR4/MyD88/NF-*κ*B signaling to facilitate PDLSC osteogenesis. Hence, the EZH2-TLR4/MyD88/NF-*κ*B axis may be an effective therapeutic candidate for periodontitis treatment.

## 2. Material and Methods

### 2.1. PDLSC Isolation and Culture

Healthy premolars were gained from the 8 patients with orthodontic teeth extraction in Beijing Shijitan Hospital. The tissues were gently removed from the middle-third root surfaces using a sterile scalpel and disaggregated, then treated with 0.2% collagenase (Gibco, Carlsbad, CA, USA) at 37°C for 2 h, and then digested with 0.25% trypsin for 30 min. Suspensions were then incubated in minimum essential medium-*α* (MEM*α*) (Gibco, Carlsbad, CA, USA) with 100 *μ*g/ml streptomycin (Beyotime, Shanghai, China), 100 U/ml penicillin (Beyotime, Shanghai, China) at 37°C in 5% CO2. All experimental protocols involving the use of these cells were approved by the Ethics Committee of The University of Beijing Shijitan Hospital.

### 2.2. Cell Transfection

Short hairpin RNA (shRNA) sequences targeting EZH2 were designed. The shRNA target EZH2 (sense: 5′-ACAUACUCUUUACUUCAUCAG-3′, antisense: 5′-GAUGAAGUAAAGAGUAUGUUU-3′) was synthesized and cloned into the pLKO.1 vector. Lentiviral knockdown EZH2 (shEZH2) particles and shNC particles were produced by GenePharma (Shanghai, China). Lipofectamine 2000 (Invitrogen) was applied to carry out plasmid transfections.

### 2.3. LPS Treatment

LPS used in this paper was purchased from Beyotime (ST1470, Shanghai, China).

### 2.4. CCK8 Assay

PDLSCs were plated into the 96-well plate in 100 *μ*l of culture medium per well at a density of 2 × 10^3^ cells 24 h prior to transfection. Between 0 and 48 h of culture, 10 *μ*l CCK8 reagent (Solarbio, Beijing, China) was added into each well and incubated for 2 h at 37°C. Relative cell proliferating rate was measured using a microplate reader (Thermo Fisher, Ltd., CA, USA) at absorbance of 450 nm.

### 2.5. Wound Healing Assay

2 × 10^5^ PDLSCs were planted in a 6-well plate and incubated for 24 h. Then, the cell monolayer was scratched using p200 pipet tip and rinsed with 1 × PBS. Then, the cells were inoculated in *α*-MEM medium enriched with 5% FBS for 48 h at 37°C in 5% CO2. The wound distance was observed using an optic Olympus BX51 microscope at light field (Olympus Corporation).

### 2.6. Immunofluorescence

PDLSCs treated with LPS, LPS + shNC, or LPS + shEZH2 were seeded on glass bottom culture dishes in a 24-well plate, fixed with 4% paraformaldehyde (Sigma) for 10 min, and perforated with 0.2% Triton-X100 for 5 min. After blocking with 10% donkey serum (Solarbio, Beijing, China) for 20 min and washing by cold 1 × PBS (Sangon Biotech, Shanghai, China), the cells were incubated with p65 (Abcam, MA, USA) at 4°C overnight. The culture dishes were incubated with AlexaFluor488-conjugated secondary antibodies (Cell Signaling Technology, Danvers, MA, USA) for 2 h. The nuclei of cells were stained with DAPI (Solarbio, Beijing, China) for 10 min. Confocal fluorescence microscopy (Zeiss Germany, Germany) was used to capture fluorescence confocal images.

### 2.7. RT-qPCR Assay

Total RNA was isolated from tissues and cells using TRIzol reagents (Thermo Fisher Scientific, USA) according to the standard protocol. cDNA was synthesized using the SuperScript IV First-Strand Synthesis System (Thermo Fisher Scientific, USA). qRT-PCR was performed with SYBR Green Master Mix (Thermo Fisher Scientific, USA). The following primer sequences were used for qRT-PCR: Runx2-Forward, 5′CTCCAACCCACGAATGCACTA3′; Runx2-Reverse, 5′GTGAGTGGGTGGCGGACATG3′; OCN-Forward, 5′GCAGAGTCCAGCAAAGGTG3′; OCN-Reverse, 5′GCCTCCTGAAAGCCGATG3′; GAPDH-Forward, 5′GGCATGGACTGTGGTCATGAG3′; GAPDH-Reverse, 5′TGCACCACCAACTGCTTAGC3′.

### 2.8. Western Blot

Cell extracts were cleaned with cold PBS buffer (Sangon Biotech, Shanghai, China), prepared with RIPA buffer (Beyotime, Shanghai, China), and supplemented with the protease inhibitor (Cocktail, MCE, USA), and protein concentrations were quantified using the BCA protein assay kit (Beyotime) according to the manufacturer's protocols. The protein lysate was separated via 10% SDS-PAGE (Solarbio, Beijing, China). Protein samples were transferred to PVDF membranes (Millipore, USA) and blocked with 5% nonfat milk (Solarbio, Beijing, China). Then, protein samples were incubated overnight at 4°C with EZH2 (CST, #5246), H3K27me3 (CST, #9733), p-p65 (CST, 3033s), p-IKB (Abcam, ab133462), TLR4 (Proteintech, 66350-1g), MyD88 (Proteintech, 23230-1-AP), Runx2 (Abcam, ab236639), OCN (Abcam, ab133612), and GAPDH primary antibodies (Cell Signaling Technology, Danvers, MA, USA). After washing, protein samples were incubated with secondary antibodies (Cell Signaling Technology, Danvers, MA, USA) for 1 h. Finally, ECL kit (Beyotime) was used to assess protein bands.

### 2.9. Alkaline Phosphatase Assays

Alkaline phosphatase (ALP) activity was assessed through an ALP assay kit (Solarbio, Beijing, China). The absorbance at 405 nm was detected by spectrophotometric methods. ALP activity was normalized by total cellular protein concentrations among the samples.

### 2.10. Alizarin Red Staining

Alizarin Red (Beyotime, Shanghai, China) staining was used to detect matrix mineralization following the manufacturer's instructions. The cells stained by alizarin red were dissolved with 10% cetylpyridinium chloride (Beyotime, Shanghai, China).

### 2.11. Flow Cytometry Analysis

For immunophenotype characterization, PDLSCs were incubated with antihuman stem cell surface-labeled antibodies including CD105-phycoerythrin, CD45-phycoerythrin, CD146-phycoerythrin, CD14-phycoerythrin, CD29-phycoerythrin, and stro1-phycoerythrin (BD Biosciences, USA). All flow cytometry tests were performed on a FACSAria (BD Bioscience).

### 2.12. Statistical Analysis

The SPSS 19.0 (IBM Corp.) statistical software was used for data analysis, and GraphPad Prism V (GraphPad Software, Inc.) was used for image editing. Measurement data were expressed as the mean ± SD and compared using the unpaired *t*-test. Differences among multiple groups were determined using one-way ANOVA with Tukey's post hoc test. *P* < 0.05 was considered to indicate a statistically significant difference.

## 3. Results

### 3.1. Periodontal Ligament Stem Cells Exhibited Stem Cell Properties

**Figure 1:**
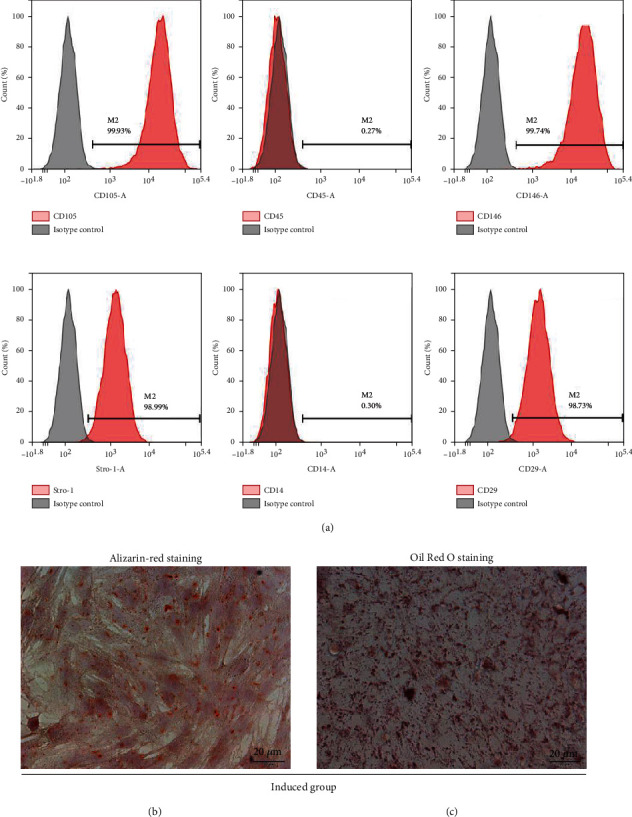
Characterization of periodontal ligament stem cells. (a) Flow cytometry results for the detection of mesenchymal stem cell markers CD105, CD45, Stro-1, CD146, CD14, and CD29 in PDLSCs. (b) Alizarin-red detection of mineralization in PDLSCs after 4 weeks induction. (c) Oil Red O staining of lipid droplets in PDLSCs after 3 weeks induction. Data are representative of three independent experiments. PDLSCs: periodontal ligament stem cells.

Flow cytometric analyses revealed high expression levels of CD146, CD29, stro-1, and CD105 but low expression levels of CD45 and CD14 (Figure 1(a)). Alizarin-red staining and Oil red O staining detected the mineralization (Figure 1(b)) and lipid droplets (Figure 1(c)) of PDLSCs induced by osteogenic and adipogenic media, respectively.

### 3.2. LPS Stimulation Significantly Promoted EZH2 and H3K27me3 Expression in PDLSCs

**Figure 2:**
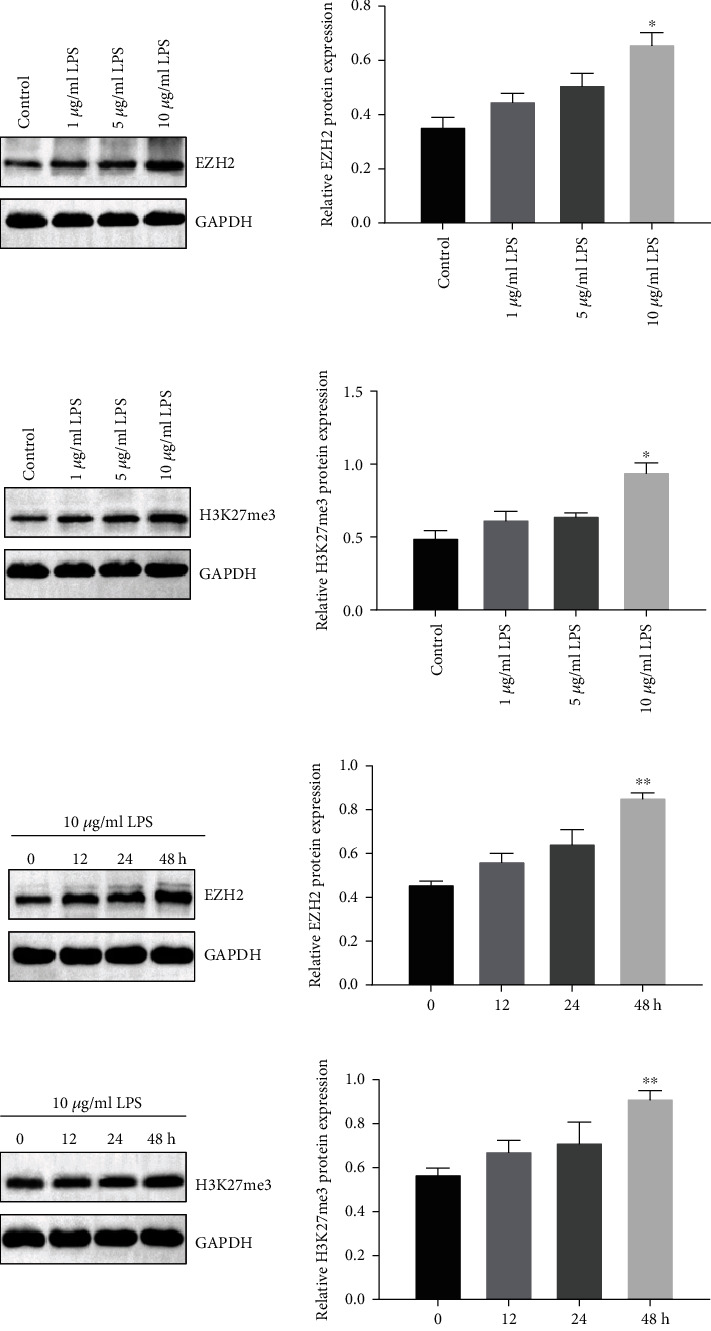
LPS stimulation promoted EZH2 and H3K27me3 expression. (a) Western blot assay showed the EZH2 protein expression in PDLSCs after the LPS stimulation in 1, 5, and 10 *μ*g/ml. (b) Western blot assay showed the H3K27me3 protein expression in PDLSCs after the LPS stimulation in 1, 5, and 10 *μ*g/ml. (c) Western blot assay showed the EZH2 protein expression in PDLSCs after 10 *μ*g/ml LPS stimulation at 12, 24, and 48 h. (d) Western blot assay showed the H3K27me3 protein expression in PDLSCs after 10 *μ*g/ml LPS stimulation at 12, 24, and 48 h. Data are representative of three independent experiments. ^∗^*P* < 0.05; ^∗∗^*P* < 0.01; ns: not significant; EZH2: enhancer of zeste 2 polycomb repressive complex 2 subunit; LPS: lipopolysaccharide.

To study the role of LPS on the EZH2 and H3K27me3 expression, we treated PDLSCs with 1, 5, and 10 *μ*g/ml LPS for 48 h. The results from western blot assay showed that 1 *μ*g/ml LPS had little effect on the basal expression of EZH2 and H3K27me3. 5 and 10 *μ*g/ml LPS obviously upregulated the expression of EZH2 and H3K27me3, and 10 *μ*g/ml LPS was selected for further study (Figures 2(a) and 2(b)). In addition, LPS treatment markedly increased the EZH2 and H3K27me3 expression levels in a time-dependent manner (Figures 2(c) and 2(d)).

### 3.3. Targeting EZH2 Diminished LPS-Induced Upregulation of TNF*α*, IL1*β*, and IL6

**Figure 3:**
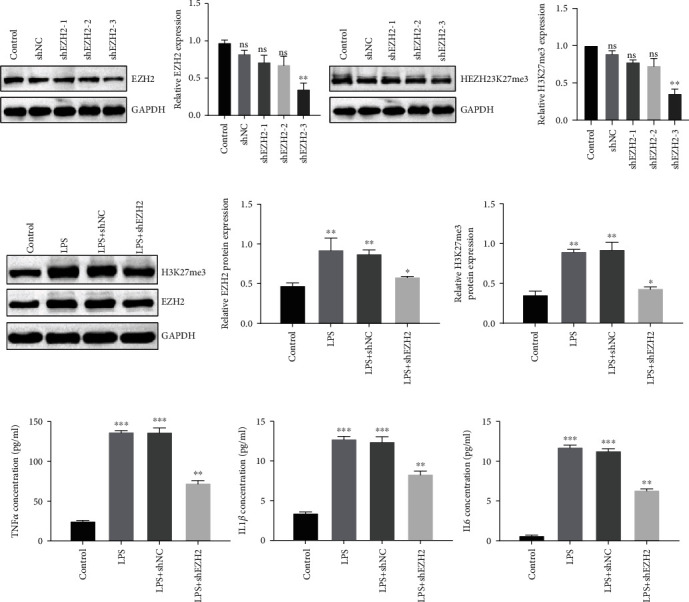
Targeting EZH2 prevented LPS-induced upregulation of TNF*α*, IL1*β*, and IL6. (a) The efficiency of shEZH2 was detected by western blot assay in PDLSCs. (b) Western blot showed the protein levels of H3K27me3 and EZH2 after LPS stimulation or LPS stimulation and shEZH2 in PDLSCs. (c) TNF*α*, IL1*β*, and IL-6 levels of PDLSCs were assessed by ELISA assays. Data are representative of three independent experiments. ^∗^*P* < 0.05; ^∗∗^*P* < 0.01; ns: not significant; EZH2: enhancer of zeste 2 polycomb repressive complex 2 subunit; LPS: lipopolysaccharide; shNC: sh-normal control; TNF*α*: tumor necrosis factor *α*; IL1*β*: interleukin 1 beta; IL6: interleukin 6.

To explore the critical role of EZH2, the shRNA was used to knockdown EZH2 expression. Western blot analysis showed the efficiency of shEZH2 in PDLSCs. The protein levels of EZH2 and H3K37me3 decreased markedly after shRNA transfection, especially in sh-EZH2-3 (Figure 3(a)). We chose sh-EZH2-3 to perform the next experiments. Western blot assay showed that EZH2 knockdown significantly inhibited LPS-induced upregulation of EZH2 and H3K27me3 expression (Figure 3(b)). The results from ELISA assay showed that EZH2 knockdown obviously reduced TNF-*α*, IL1*β*, and IL-6 levels in supernatant after LPS stimulation (Figure 3(c)).

### 3.4. Targeting EZH2 Reduced LPS-Induced Proliferation and Migration Inhibition

**Figure 4:**
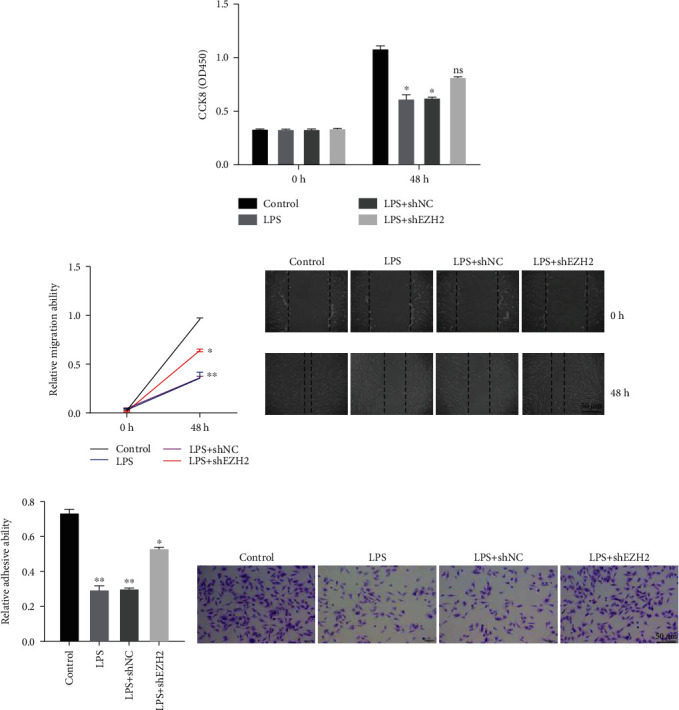
Targeting EZH2 reduced LPS-induced proliferation and migration inhibition. (a) The role of altered EZH2 expression on PDLSC cellular viabilities was detected by CCK8 assay. (b) The role of altered EZH2 expression on cell migration abilities was detected by wound healing assay. (c) The role of altered EZH2 expression on cell adhesion abilities was detected by cell adhesion assay. Data are representative of three independent experiments. ^∗^*P* < 0.05; ^∗∗^*P* < 0.01; ns: not significant; LPS: lipopolysaccharide; shNC: sh-normal control; h: hour; CCK8: cell counting kit 8.

To further realize the role of EZH2 in periodontitis progression, the viability and migration of PDLSCs were determined by CCK8, wound healing, and adhesion assays, respectively. The results from CCK8 assay indicated that the LPS challenge decreased cell viability and shEZH2 reduced LPS-induced proliferation inhibition (Figure 4(a)). In addition, the results from the wound healing assay suggested that the LPS challenge inhibited cell migration and shEZH2 reduced LPS-induced migration inhibition (Figure 4(b)). Moreover, the results from the cell adhesion assay suggested that the LPS challenge inhibited cell adhesion and shEZH2 reduced LPS-induced adhesion inhibition (Figure 4(c)).

### 3.5. EZH2 Knockdown Inhibits TLR4/MyD88/NF-*κ*B Signaling to Facilitate PDLSC Osteogenesis

**Figure 5:**
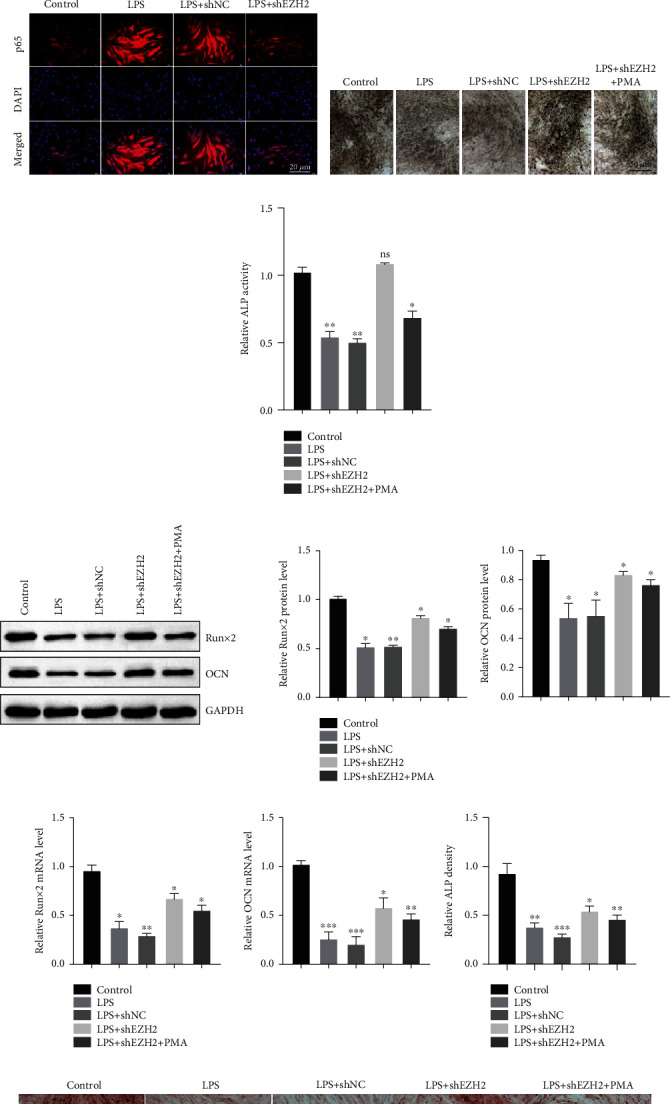
EZH2 knockdown inhibits TLR4/MyD88/NF-*κ*B signaling to facilitate PDLSC osteogenesis. (a) Western blot showed the phosphorylated p65, phosphorylated IKB, TLR4, and MyD88 protein level transfected with the indicated plasmids in PDLSCs. (b) IF showed the distribution of p65 in the indicated treatment. (c, d) ALP activity in PDLSCs after the indicated treatments. (e) Western blot showed the Runx2 and OCN protein level after the indicated treatment in PDLSCs. (f) qPCR showed the mRNA level of Runx2 and OCN. (g, h) Alizarin red staining showed the role of EZH2 and LPS on the mineralization in PDLSCs. Data are representative of three independent experiments. ^∗^*P* < 0.05; ^∗∗^*P* < 0.01; ns: not significant; LPS: lipopolysaccharide; shNC: sh-normal control; p-p65: phosphorylated p65; p-IKB: phosphorylated IKB; TLR4: toll-like receptor 4; MyD88: myeloid differentiation primary response gene 88; PMA: phorbol ester; ALP: alkaline phosphatase assay; OCN: bone gamma-carboxyglutamate protein; Runx2: RUNX family transcription factor 2.

Western blot assay showed that phosphorylated p65 and IKB, TLR4, and MyD88 were found to be upregulated after LPS stimulation. shEZH2 partly reversed the effect of LPS on the expression of phosphorylated p65 and IKB, TLR4, and MyD88 (Figure 5(a)). Immunofluorescence assay showed that LPS increased the nuclear distribution of p65 and shEZH2 partly the effect of LPS on the distribution of p65 in PDLSCs (Figure 5(b)).

LPS inhibited PDLSC osteogenesis as indicated by the reduced ALP activity, and EZH2 knockdown by shEZH2 significantly enhanced ALP activity (Figures 5(c) and 5(d)). Western blot showed that LPS significantly inhibited RUNX2 and OCN protein expression levels. EZH2 knockdown by shEZH2 significantly enhanced RUNX2 and OCN protein levels compared with the LPS treatment only (Figure 5(e)). To verify whether EZH2 regulated PDLSC osteogenic differentiation through NF-*κ*B signaling, cells were pretreated with the NF-*κ*B activator, PMA. PMA could mitigate PDLSC osteogenic differentiation in inflammatory microenvironments following the restrained Runx2 and OCN protein levels (Figure 5(e)). The results from qPCR showed the similar trend in PDLSCs (Figure 5(f)). And that, EZH2 knockdown enhanced PDLSC osteogenesis as indicated by the accumulated mineralization using alizarin red staining (Figures 5(g) and 5(h)), indicating that EZH2 regulates PDLSC osteogenesis through the NF-*κ*B signaling pathway.

## 4. Discussion

Periodontitis induced by bacteria especially Gram-negative bacteria is the most prevalent chronic inflammatory disease worldwide [15]. Infections and the subsequent host immune responses have remodeled an aberrant microenvironment, which generally lead to the dysfunction of PDLSCs, which are the potential “seed cells” for periodontal regeneration applications and bone tissues engineering [16, 17]. Interestingly, recent studies have reported that scathing PDLSCs may conduce to the perturbation of periodontal homeostasis [18–20].

Various mediators, including cytokines, chemokines and the inducible synthesized enzymes, regulate the occurrence and extinction of inflammation [21]. Many papers have reported that the histone-lysine N-methyltransferase EZH2 regulated a wide range of biological processes, including cell senescence, stem cell renewal, tumor progression, and immune response, which mediates gene silencing by mediating the trimethylation of H3K27me3 through the regulation in promoter region [22–24]. Wang et al. reported that inhibition of EZH2 alleviated P. acnes plus LPS-induced fulminant hepatic failure (FHF) by repressing RUNX1 in dendritic cells [25]. LPS/ATP-induced MALAT1 upregulation leads to the increased ROS levels and inflammasome activation by regulating EZH2-mediated epigenetic repression in Parkinson's disease (PD) [26]. In addition, Jing et al. reported that EZH2 expression was obviously enhanced in osteoporotic MSCs and DZNep, an inhibitor of the H3K27me3, effectively augmented Wnt signaling and improved osteogenic differentiation of osteoporotic MSCs in vitro [27]. Here, we reported that LPS stimulation significantly promoted EZH2 and H3K27me3 expression in PDLSCs in a time-dependent and dose-dependent manner.

LPS stimulation mainly augmented the expression levels of proinflammatory cytokines, including interleukin IL-1 beta (IL1*β*), tumor necrosis factor-alpha (TNF*α*), and IL-6 [28, 29], resulting in an inflammatory response and destruction of periodontal tissue during periodontitis [30]. We found that EZH2 knockdown significantly downregulated TNF-*α*, IL1*β*, and IL-6 levels in supernatant after osteoblast induction with LPS stimulation in PDLSCs. In addition, we found that LPS stimulation attenuated cell proliferation, cell migration, and cell adhesion in PDLSCs. However, EZH2 knockdown significantly rescued the inhibition of cell proliferation, cell migration, and cell adhesion by LPS stimulation in PDLSCs.

The hallmark of inflammatory response is a stronger activation of the NF-*κ*B pathway [31]. The NF-*κ*B family controls many important regulatory genes such as immunity-related, cell death-related, inflammation-related, and cell proliferation-related and plays an important role in the cellular environment [32, 33]. A key function of NF-*κ*B is controlling the immune response at various stages, including innate immune response and adaptive immune response [34]. Wang et al. reported that LPS, a pivotal periodontal pathogenic factor, activates the NF-*κ*B signaling pathway and augments the inflammatory cytokine release [35]. TLR4 participated in the induced expression of NF-*κ*B protein after LPS stimulation [36]. Hence, we reported that LPS stimulation upregulated the phosphorylated p65 and IKB, TLR4, and MyD88, and shEZH2 partly reversed the effect of LPS on the expression of phosphorylated p65 and IKB, TLR4, and MyD88. In addition, EZH2 affected MSC osteogenesis by directly regulating lineage-specific transcription factors and the signaling modules as well as many novel genes. Administration of the EZH2 inhibitor GSK126 enhanced osteoblast differentiation in MC3T3 mice, following by the enhanced mineralized nodules stained with alizarin red and the increased alkaline phosphatase activity. Inhibition of EZH2 ameliorated the BMP2-mediated osteoblast differentiation of the progenitor cells and induction of stereotyped osteoblast maturation [37]. Osteogenic regeneration is an important process of the periodontal tissue regeneration, and many studies have shown that the osteogenic differentiation ability of PDLSCs is significantly reduced under the inflammatory conditions [38, 39].

In this study, we found that LPS significantly inhibited Runx2, OCN levels, and ALP activity, which are the marker genes of osteogenesis [30]. EZH2 knockdown by shEZH2 significantly enhanced RUNX2 and OCN levels and ALP activity compared with the LPS group.

In conclusion, we confirmed that EZH2 regulated cell proliferation, cell migration, inflammatory factor release, and osteogenesis in PDLSCs. EZH2 knockdown rescued the phenotypes induced by LPS through regulating the TLR4/MyD88/NF-*κ*B signaling pathway. This may also provide new therapeutics for periodontitis.

## Data Availability

The data used to support the findings of this study are available from the corresponding author upon request.
